# Prognostic significance of pretreatment peripheral blood inflammatory biomarkers in elderly patients with locally advanced cervical cancer undergoing definitive chemoradiotherapy

**DOI:** 10.3389/fonc.2026.1935344

**Published:** 2026-09-03

**Authors:** Xiuhua Li, Jianjian Qiu, Ting Lin, Yuling Ye

**Affiliations:** 1 Department of Gynecology, Clinical Oncology School of Fujian Medical University, Fujian Cancer Hospital, Fuzhou, Fujian, China; 2 Department of Radiation Oncology, Clinical Oncology School of Fujian Medical University, Fujian Cancer Hospital, Fuzhou, Fujian, China

**Keywords:** definitive chemoradiotherapy, elderly locally advanced cervical squamous cell carcinoma, NLR, recursive partitioning analysis, SIRI

## Abstract

**Background:**

Systemic inflammatory response plays a critical role in tumor progression and immune evasion. Elderly patients with locally advanced cervical squamous cell carcinoma (CSCC) receiving definitive chemoradiotherapy (dCRT) exhibit high prognostic heterogeneity, and there is a lack of simple and effective risk stratification tools. This study aimed to evaluate the prognostic value of pretreatment peripheral blood inflammatory biomarkers and to construct a recursive partitioning analysis (RPA) risk stratification mode.

**Methods:**

A total of 441 elderly patients with locally advanced CSCC who received dCRT were included. Variable selection was performed using LASSO regression, independent prognostic factors were identified via multivariate Cox regression, and an RPA model was constructed. The performance of the final RPA model was internally validated using bootstrap resampling (1,000 resamples) and 10-fold cross-validation, with the concordance index (C-index), area under the curve (AUC), calibration curves, and decision curve analysis (DCA) used as evaluation metrics.

**Results:**

LASSO regression identified seven potential predictive factors from 18 candidate variables. Multivariable Cox regression further confirmed that NLR and SIRI were independent prognostic factors for overall survival and progression-free survival, and SHAP visualization analysis further validated their core contributions. Elevated NLR and SIRI levels were significantly associated with larger tumor diameter, higher FIGO stage, and poorer prognosis. Restricted cubic spline analysis demonstrated a linear association between NLR/SIRI and OS as well as PFS. A RPA risk stratification model based on NLR, SIRI, and FIGO stage classified patients into low-risk (5-year OS rate: 82.1%), intermediate-risk (70.0%), and high-risk (28.6%) groups. This model showed superior predictive performance compared to traditional FIGO stage and individual inflammatory biomarkers and remained robust across all subgroups.

**Conclusion:**

Pretreatment NLR and SIRI are independent prognostic biomarkers for survival in elderly patients with locally advanced CSCC undergoing dCRT. The RPA model, which integrates NLR, SIRI and FIGO stage, can be used as an auxiliary prognostic stratification tool, but further external validation is required.

## Introduction

Cervical cancer (CC) is one of the most common malignant tumors among women worldwide, with incidence and mortality rates remaining high in developing countries (1). In China, approximately 109,741 new cases of CC are reported annually, of which 24.8% occur in patients aged 60 years or older, and deaths among those aged ≥60 account for 45.07% of all CC-related deaths (2, 3). With the accelerating trend of population aging, the diagnosis and treatment of elderly patients with CC have become increasingly prominent (4). Although surgery is the primary treatment for early-stage CC, most elderly patients are often unable to tolerate radical surgery due to comorbidities, poor performance status, or diagnosis at a locally advanced stage. Definitive chemoradiotherapy (dCRT), as the standard treatment for locally advanced CC, is also widely applied in the elderly population (5). However, the tolerance and prognosis of elderly patients receiving dCRT are highly heterogeneous, and there is a lack of simple and effective biomarkers to identify high-risk individuals early and guide personalized treatment, representing an urgent clinical problem to be addressed.

In recent years, the role of systemic inflammatory response in tumor initiation, progression, and treatment resistance has attracted widespread attention. Numerous studies have confirmed that inflammatory cells within the tumor microenvironment and their secreted cytokines can accelerate tumor progression through mechanisms such as promoting angiogenesis, inhibiting apoptosis, and inducing immune evasion (6, 7). Peripheral blood-derived inflammatory indicators, such as the neutrophil-to-lymphocyte ratio (NLR), platelet-to-lymphocyte ratio (PLR), lymphocyte-to-monocyte ratio (LMR), derived neutrophil-to-lymphocyte ratio (dNLR), prognostic nutritional index (PNI), platelet-to-albumin ratio (PAR), systemic immune-inflammation index (SII), and systemic inflammation response index (SIRI), are easy to measure, cost-effective, and reproducible. These indicators have been demonstrated to have significant prognostic value in various solid tumors, including lung cancer, colorectal cancer, and gastric cancer (8–14). In the field of cervical squamous cell carcinoma (CSCC), previous studies have preliminarily explored the prognostic significance of these indicators in patients treated with surgery or radiotherapy alone (15); however, systematic evaluations focusing specifically on elderly patients who received dCRT remain limited.

Elderly patients often exhibit declined immune function and bone marrow reserve capacity, and their inflammatory response patterns may differ from those of non-elderly patients. Therefore, this study retrospectively analyzed clinical data from elderly patients with cervical cancer who underwent dCRT. Using LASSO regression for variable selection, multivariate Cox regression to identify independent prognostic factors, and SHapley Additive exPlanations (SHAP) for visual interpretation, we systematically evaluated the associations between pretreatment inflammatory indicators and overall survival (OS) and progression-free survival (PFS). The aim was to clarify the prognostic predictive value of these indicators in this special population and provide a reference for clinical decision-making.

## Materials and methods

### Patients

This study retrospectively analyzed the clinical data of 441 elderly patients with locally advanced CSCC who received treatment at Fujian Cancer Hospital from January 2017 to December 2022. The inclusion criteria were as follows: (1) age ≥60 years at diagnosis; (2) histopathologically confirmed CSCC; (3) receipt of dCRT; (4) clinical stage IB3–IVA according to the International Federation of Gynecology and Obstetrics (FIGO) classification; (5) treatment-naïve patients who had not previously undergone hysterectomy, radiotherapy, or systemic antitumor therapy; and (6) complete pretreatment peripheral blood routine data. The exclusion criteria were: (1) presence of other primary malignant tumors; (2) acute infection or chronic inflammatory disease within 2 weeks before treatment; (3) hematological disorders or immunodeficiency; (4) systemic use of glucocorticoids, immunosuppressants, or granulocyte colony-stimulating factor within 4 weeks before treatment; and (5) missing clinical data. This study was conducted in accordance with the principles of the Declaration of Helsinki and was approved by the Ethics Committee of Fujian Cancer Hospital (K2026-297-01).

### Data collection

Through the hospital electronic medical record system, clinical data were retrospectively collected from all patients meeting the inclusion criteria, including demographic and baseline characteristics (age, body mass index (BMI)), tumor-related characteristics (FIGO stage, maximum tumor diameter, lymph node metastasis status), and treatment information (radiation dose, chemotherapy regimen and cycles). For laboratory indicators, peripheral blood routine data (neutrophil, lymphocyte, platelet, and monocyte counts) and biochemical indicators (albumin, lactate dehydrogenase) were collected within one week prior to treatment. Based on these data, the following inflammatory markers were calculated: PNI = albumin (g/L) + 5 × lymphocyte count (×10^9^/L), PAR = platelet count/albumin, PLR = platelet count/lymphocyte count, NLR = neutrophil count/lymphocyte count, dNLR = neutrophil count/(white blood cell count − neutrophil count), LMR = lymphocyte count/monocyte count, SII = (neutrophil count × platelet count)/lymphocyte count, and SIRI = (neutrophil count × monocyte count)/lymphocyte count.

### Treatment

All patients received dCRT. Radiotherapy consisted of pelvic external beam radiation therapy (EBRT) using three-dimensional conformal or intensity-modulated techniques (45–50.4 Gy in 25–28 fractions) combined with high-dose-rate brachytherapy, delivering a cumulative dose to point A ≥ 85 Gy (EQD2). Concurrent chemotherapy was primarily platinum-based combined with taxane regimens. During treatment, doses were adjusted according to toxicity, and radiotherapy plans were verified by physics quality assurance with regular cone-beam computed tomography (CBCT) for setup verification.

### Study endpoints and follow-up

The primary endpoints of this study were OS and PFS. OS was defined as the time from pathological diagnosis to death from any cause or the last follow-up date, while PFS was defined as the time from pathological diagnosis to the first documented disease progression, recurrence, death, or the last follow-up date. Follow-up was conducted after treatment completion, with visits every 3 months for the first 2 years, every 6 months from year 3 to 5, and annually thereafter. The follow-up cutoff date was December 2025. Each follow-up visit included medical history taking, physical examination (including gynecological examination), serum tumor marker testing (e.g., squamous cell carcinoma antigen), and pelvic MRI or CT assessment. Chest CT, PET-CT, or pathological biopsy was performed when necessary to confirm recurrence or metastasis.

### Statistical analysis

We performed statistical analyses using R software (version 4.5.2) and SPSS (version 25.0). Optimal cut-off values for age, BMI, radiation dose, and pretreatment inflammatory markers were determined using receiver operating characteristic (ROC) curve analysis with the maximum Youden index. Variable selection was then performed using least absolute shrinkage and selection operator (LASSO) regression with 10-fold cross-validation, and the penalty parameter λ was chosen based on the minimum partial likelihood deviance. The selected variables were subsequently entered into multivariate Cox proportional hazards regression to identify independent prognostic factors. To assess multicollinearity among the included variables, the variance inflation factor (VIF) was calculated, with a VIF > 10 indicating the presence of multicollinearity. Finally, the SHapley Additive exPlanations (SHAP) method was used to visualize the contribution of each independent prognostic factor to the model’s prediction. Based on the NLR, SIRI, and FIGO stage, a risk stratification model was constructed using recursive partitioning analysis (RPA) for patient prediction and classification. Internal validation was performed using bootstrap resampling (1,000 resamples) and 10-fold cross-validation, and the concordance index (C-index) was calculated. The performance of the RPA model was evaluated using ROC curves, decision curve analysis (DCA), and calibration curves. Net reclassification improvement (NRI) and integrated discrimination improvement (IDI) were calculated to quantify the incremental predictive value of the RPA model over FIGO stage alone, with 95% confidence intervals derived from 1000 bootstrap resamples. All tests were two-tailed, and a P-value < 0.05 was considered statistically significant.

## Results

### Patient characteristics

This study included 441 eligible elderly patients with locally advanced CSCC. The baseline characteristics of the patients were as follows (**Table 1**): 260 patients (59.0%) were aged ≥66 years; 250 patients (56.7%) had FIGO stage III/IV disease, 150 (34.0%) had a maximum tumor diameter ≥4.90 cm, and 85 (19.3%) had positive lymph node metastasis. Regarding treatment, 306 patients (69.4%) received pelvic EBRT with a dose ≥48.05 Gy, 69 (15.6%) received brachytherapy dose ≥56.44 Gy, 252 (57.1%) received a total radiotherapy dose ≥88.27 Gy, and 227 (51.5%) received ≥2 cycles of chemotherapy. The optimal cutoff values for BMI, PNI, PAR, PLR, NLR, dNLR, LMR, SII, and SIRI were 21.70, 50.35, 8.74, 132.31, 2.77, 2.08, 4.75, 811.84, and 1.42, respectively. Additionally, 182 patients (41.3%) had lactate dehydrogenase (LDH) levels ≥190 U/L.

**Table 1:** Patient baseline characteristics of locally advanced elderly CSCC.

Variables	No. of patients
Age (year)	
<66	181 (41.0%)
≥81	260 (59.0%)
FIGO Stage	
I/II	191 (43.3%)
III/IV	250 (56.7%)
Tumor diameter (cm)	
<4.90	291 (66.0%)
≥91(6	150 (34.0%)
Lymphatic metastasis	
No	356 (80.7%)
Yes	85 (19.3%)
EBRT (Gy)	
<48.05	135 (30.6%)
≥35(30	306 (69.4%)
Brachytherapy dose (Gy)	
<56.44	372 (84.4%)
≥72(84	69 (15.6%)
Total dose (Gy)	
<88.27	189 (42.9%)
≥89(42	252 (57.1%)
Chemotherapy cycles	
<2	214 (48.5%)
≥1	227 (51.5%)
BMI	
<21.70	163 (37.0%)
≥63(37	278 (63.0%)
PNI	
<50.35	180 (40.8%)
≥80(40	261 (59.2%)
PAR	
<8.74	387 (87.8%)
≥87(8	54 (12.2%)
PLR	
<132.31	249 (56.5%)
≥49(56.	192 (33.5%)
NLR	
<2.77	358 (81.2%)
≥58(8	83 (18.8%)
dNLR	
<2.08	349 (79.1%)
≥49(7	92 (20.9%)
LMR	
<4.75	154 (34.9%)
≥54(3	287 (65.1%)
SII	
<811.84	356 (80.7%)
≥56(80.	86 (19.3%)
SIRI	
<1.42	384 (87.0%)
≥84(8	57 (13.0%)
LDH	
<190	259 (58.7%)
≥59(	182 (41.3%)

FIGO, International Federation of Gynecology and Obstetrics; EBRT, external beam radiotherapy; BMI, body mass index; PNI, prognostic nutritional index; PAR, platelet-to-albumin ratio; PLR, platelet-to-lymphocyte ratio; NLR, neutrophil-to-lymphocyte ratio; dNLR, derived neutrophil-to-lymphocyte ratio; LMR, lymphocyte-to-monocyte ratio, SII, systemic immune-inflammation index; SIRI, systemic inflammation response index.

### LASSO regression, multivariate Cox regression analysis, and SHAP visualization

**Figure 1:**
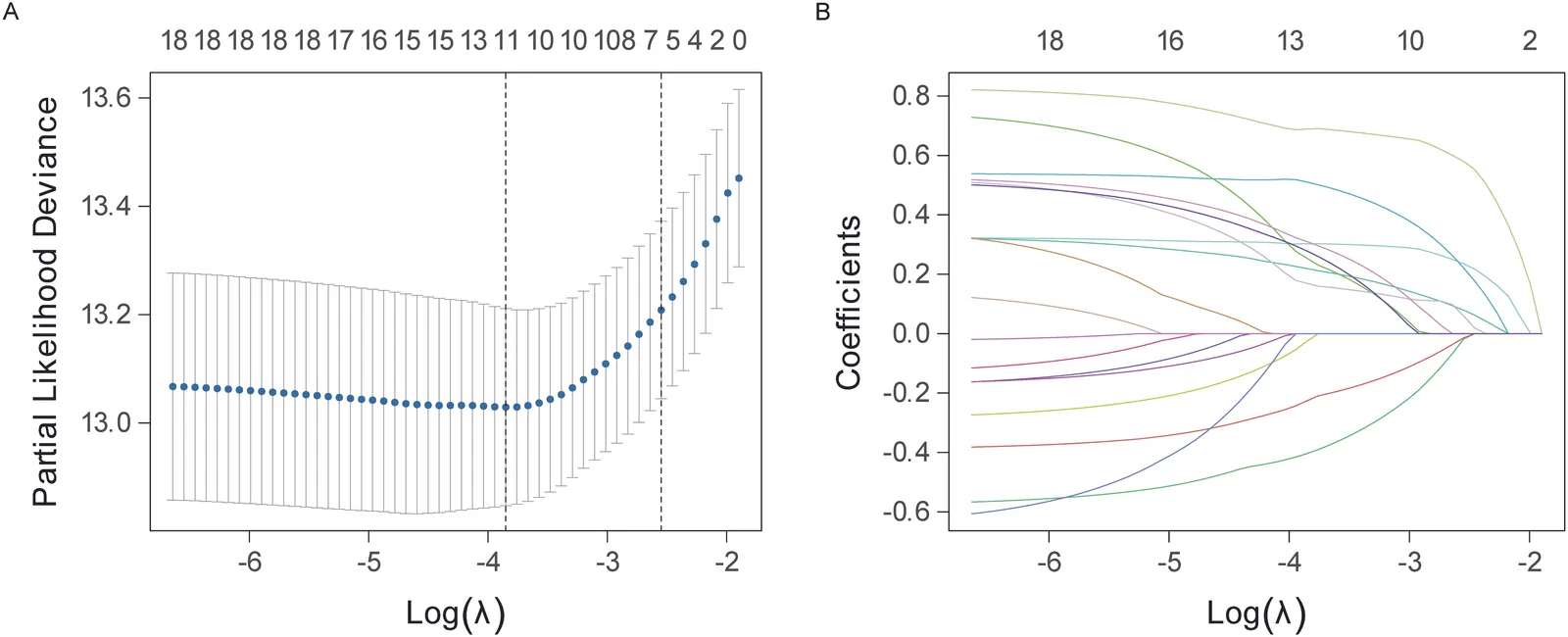
LASSO regression to reduce dimensionality among the 18 candidate variables. **(A)** Prognostic LASSO coefficient selection and **(B)** variable trajectory.

**Figure 2:**
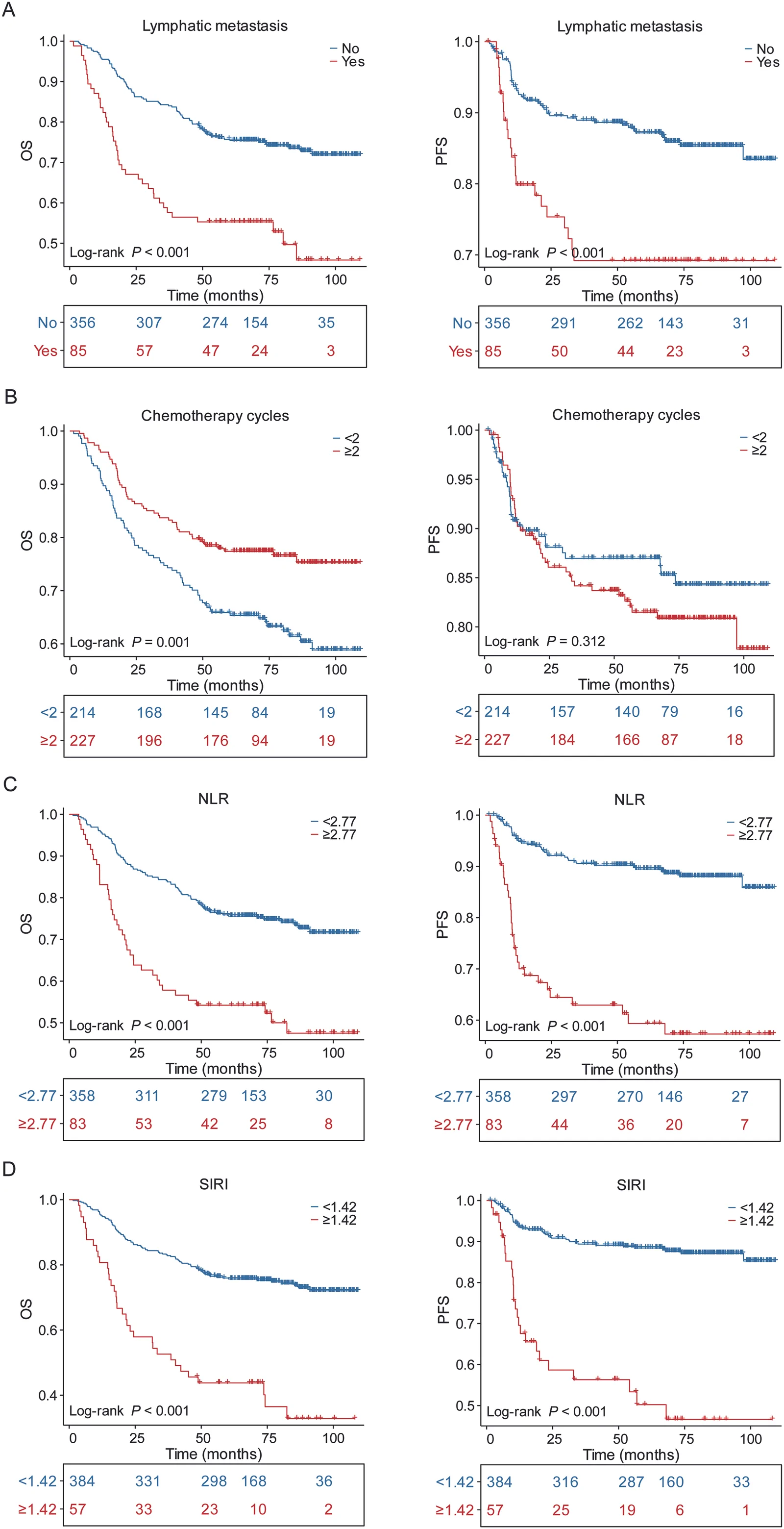
Kaplan-Meier survival curve of independent prognostic factors. **(A)** Lymphatic metastasis; **(B)** Chemotherapy cycles; **(C)** NLR; **(D)** SIRI.

We performed LASSO regression to reduce dimensionality among the 18 candidate variables, ultimately identifying seven variables with non-zero coefficients (**Figure 1A**), and illustrated the coefficient shrinkage process using a variable trajectory plot. VIF analysis confirmed the absence of multicollinearity among these variables (**Figure 1B**). Subsequently, the seven selected variables were entered into multivariate Cox regression analysis. The results showed that lymphatic metastasis (HR = 1.999, 95% CI 1.283-3.114, P = 0.002), chemotherapy cycles (HR = 0.546, 95% CI 0.383-0.779, P = 0.001), NLR (HR = 1.624, 95% CI 1.055-2.499, P = 0.028), and SIRI (HR = 2.187, 95% CI 1.406-3.404, P = 0.001) were independent prognostic factors for OS, whereas only NLR (HR = 2.491, 95% CI 1.437-4.318, P = 0.001) and SIRI (HR = 2.764, 95% CI 1.538-4.964, P = 0.001) were independent prognostic factors for PFS (**Table 2**). The Kaplan-Meier survival curve demonstrates significant differences in OS and PFS based on independent prognostic factors (**Figure 2**).

**Table 2:** Multivariate Cox regression analysis for OS and PFS.

	OS		PFS	
Variables	HR (95% CI)	P value	HR (95% CI)	P value
FIGO Stage				
III/IV VS. I/II	1.252 (0.866-1.817)	0.231	1.198 (0.729-1.968)	0.475
Tumor diameter (cm)				
≥cm)e VS. <4.90	1.373 (0.933-2.020)	0.108	1.382 (0.809-2.361)	0.236
Lymphatic metastasis				
Yes VS. No	1.999 (1.283-3.114)	0.002	1.738 (0.945-3.197)	0.075
Chemotherapy cycles				
≥y VS. <2	0.546 (0.383-0.779)	0.001	1.256 (0.764-2.064)	0.368
PNI				
≥NI68(VS. <50.35	0.780 (0.541-1.124)	0.183	0.605 (0.359-1.021)	0.060
NLR				
≥LR60 VS. <2.77	1.624 (1.055-2.499)	0.028	2.491 (1.437-4.318)	0.001
SIRI				
≥IRI1 VS. <1.42	2.187 (1.406-3.404)	0.001	2.764 (1.538-4.964)	0.001

FIGO, International Federation of Gynecology and Obstetrics; PNI, prognostic nutritional index; NLR, neutrophil-to-lymphocyte ratio; SIRI, systemic inflammation response index.

**Figure 3:**
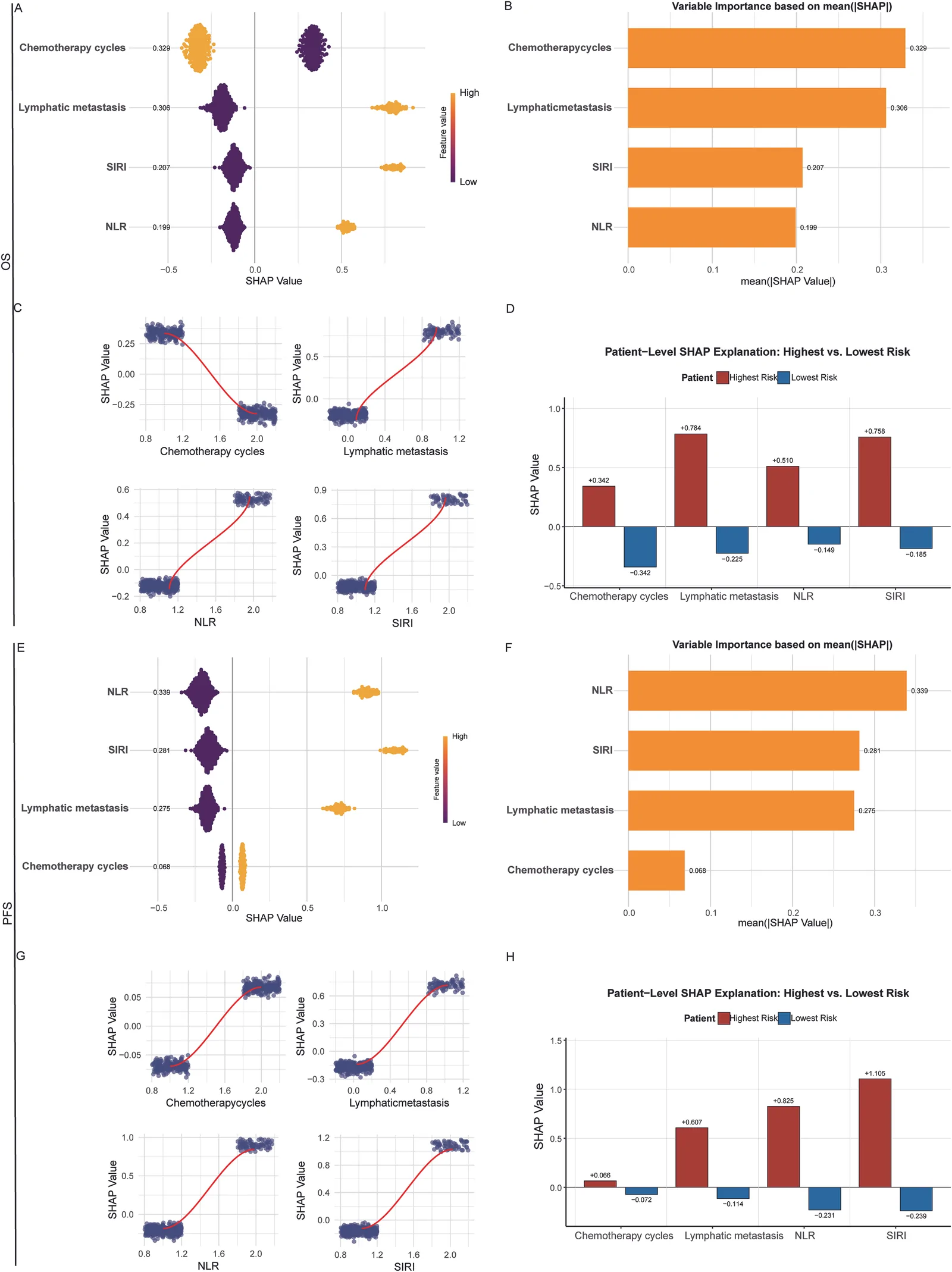
SHAP visualization: **(A, E)** beeswarm plot for OS and PFS; **(B, F)** feature importance bar plot; **(C, G)** dependence plot; **(D, H)** force plot.

SHAP swarm plots revealed that higher levels of NLR and SIRI, as well as the presence of lymphatic metastasis, were associated with positive SHAP values (indicating increased risk of death or disease progression) (**Figures 3A, E**). SHAP feature importance analysis demonstrated that the mean absolute SHAP values of pretreatment NLR and SIRI were relatively high, suggesting that these two variables contributed substantially to the predictive model (**Figures 3B, F**). Dependence plots further revealed that SHAP values increased sharply once NLR and SIRI exceeded their optimal cutoff values, confirming their nonlinear effects (**Figures 3C, G**). In the force plot for the highest-risk patient, elevated SIRI, high NLR, and positive lymphatic metastasis collectively increased the predicted risk; conversely, the lowest-risk patient exhibited the opposite pattern, indicating a favorable prognosis (**Figures 3D, H**). Collectively, SHAP analysis identified NLR and SIRI as core prognostic factors and intuitively illustrated the contribution of each variable to individual risk prediction.

### The relationship between NLR, SIRI and the clinical characteristics of patients

**Figure 4:**
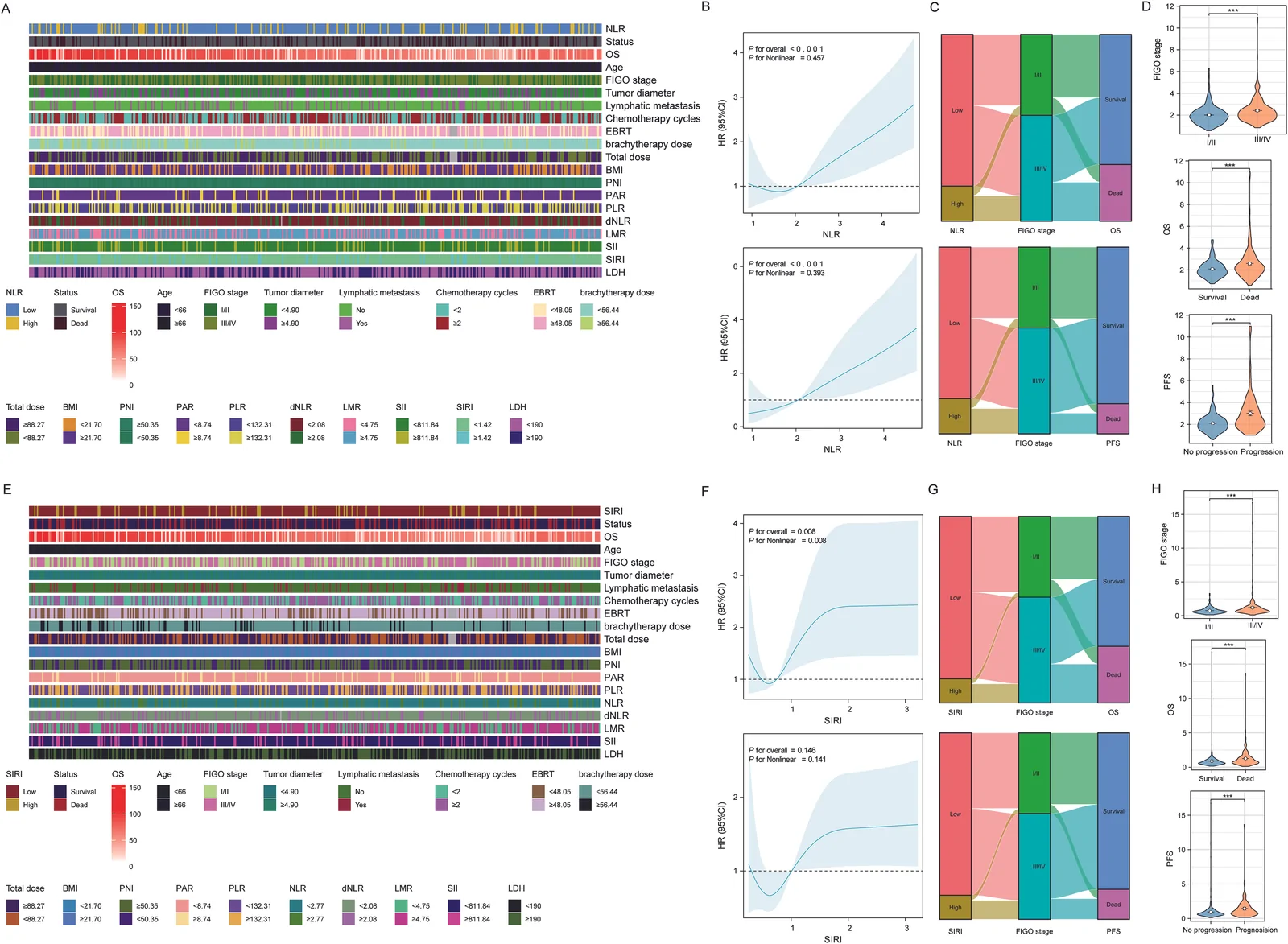
Heatmap of clinical characteristics distribution in the high- and low-NLR/SIRI groups **(A, E)**; RCS analysis revealing linear relationships between NLR, SIRI, and survival outcomes **(B, F)**; Sankey diagram showing the relationships among NLR, SIRI, FIGO stage, and survival outcomes **(C, G)**; violin plots showing FIGO stage and survival outcomes **(D, H)**.

The heatmaps in **Figures 4A, E** illustrated differences in the distribution of clinical characteristics between the high and low NLR and SIRI groups: high levels of NLR and SIRI were significantly associated with larger tumor diameter, higher FIGO stage, and poorer prognosis. Restricted cubic spline (RCS) analysis demonstrated that NLR and SIRI were linearly associated with OS and PFS (**Figures 4B, F**). Sankey diagrams further elucidated the interrelationships among NLR, SIRI, FIGO stage, OS, and PFS (**Figures 4C, G**). Violin plots revealed significantly higher inflammatory levels, as reflected by NLR and SIRI, in patients who experienced death, disease progression, or had a higher FIGO stage (**Figures 4D, H**).

### Construct an RPA model based on NLR, SIRI, and FIGO staging

**Figure 5:**
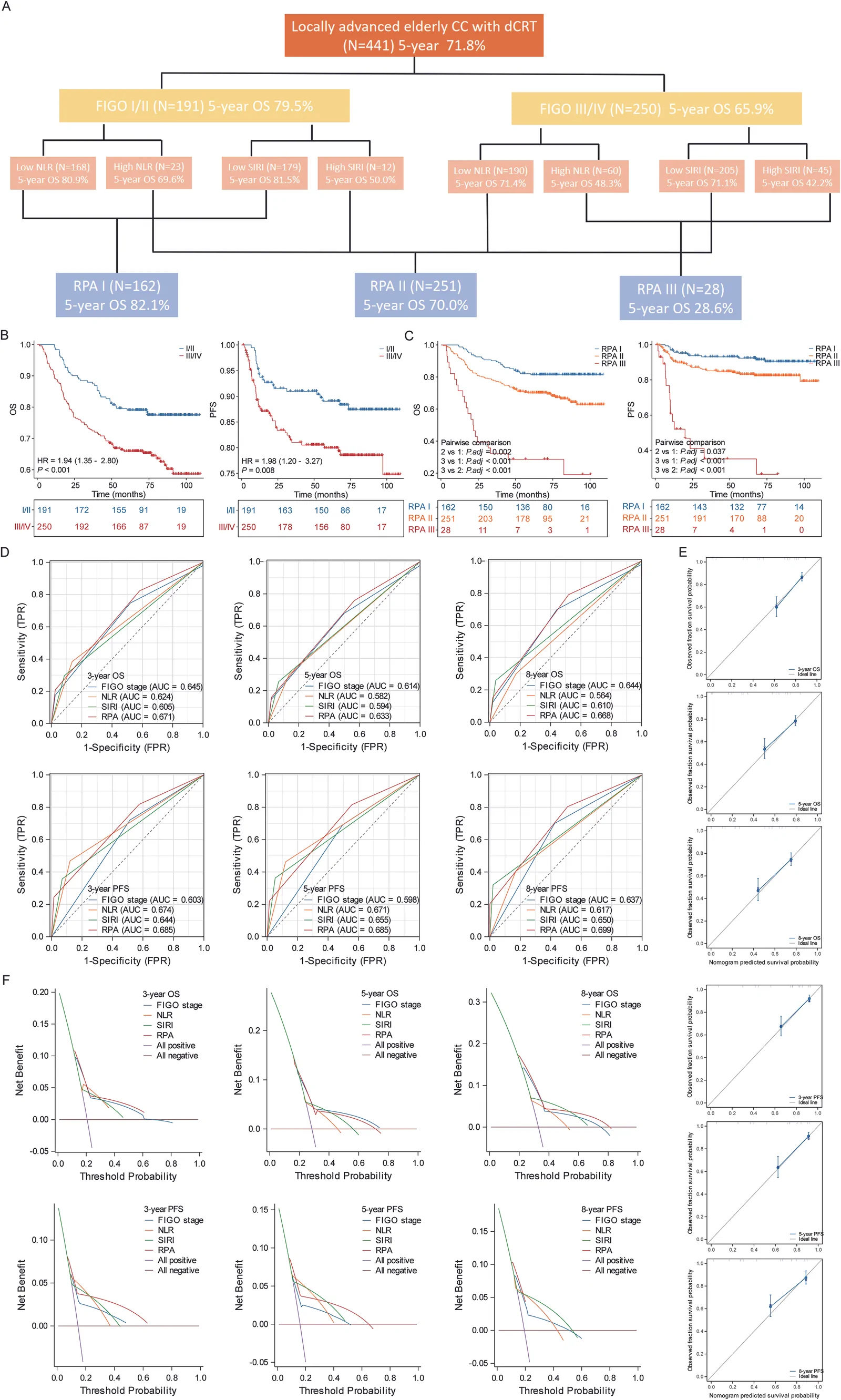
**(A)** The RPA risk model integrating NLR, SIRI, and FIGO staging; **(B, C)** survival curves based on FIGO staging and RPA stratification; **(D)** ROC; **(E)** calibration curve; **(F)** DCA.

Based on NLR, SIRI, and FIGO stage, a RPA risk stratification model was constructed. This model classified 441 elderly patients with locally advanced CSCC into three risk groups: RPA Group I (low NLR, low SIRI, and FIGO stage I/II, n = 162), RPA Group II (high NLR, high SIRI, and FIGO stage I/II, or low NLR, low SIRI, and FIGO stage III/IV, n = 251), and RPA Group III (high NLR, high SIRI, and FIGO stage III/IV, n = 28). The 5-year OS rates for Groups I, II, and III were 82.1%, 70.0%, and 28.6%, respectively. The 5-year OS rates for patients with FIGO stage I/II and III/IV were 79.5% and 65.9%, respectively. Compared with traditional FIGO staging, the RPA model demonstrated superior risk discrimination (**Figure 5A**). Kaplan–Meier survival curves further confirmed that risk stratification based on the RPA model outperformed FIGO staging in predicting patient prognosis (**Figures 5B, C**). The area under the curve values for the RPA model in predicting 3-, 5-, and 8-year OS and PFS were all higher than those of FIGO staging and individual NLR and SIRI (**Figure 5D**). Calibration curves showed good agreement between the predicted probabilities and actual observations (**Figure 5E**). Furthermore, DCA indicated that the RPA model provided greater clinical net benefit (**Figure 5F**).

### Model comparison

We compared the discriminative performance of three competing models—FIGO stage alone, NLR+SIRI alone, and the full RPA model—for both OS and PFS. For OS, the C-index was 0.625 (95% CI 0.583-0.667) for the RPA model, 0.580 (95% CI 0.540-0.619) for FIGO stage alone, and 0.605 (95% CI 0.563-0.647) for NLR+SIRI alone. For PFS, the corresponding C-index values were 0.663 (95% CI 0.606-0.721), 0.584 (95% CI 0.530–0.638), and 0.688 (95% CI 0.629–0.746), respectively. Compared with FIGO stage alone, the RPA model demonstrated significant incremental value for OS [IDI = 0.072 (0.026-0.136) at 3 years and 0.063 (0.023-0.114) at 5 years; continuous NRI = 0.394 (0.192-0.603) and 0.308 (0.145–0.484), respectively] and for PFS [IDI = 0.114 (0.045–0.216) and 0.113 (0.046-0.206); continuous NRI = 0.536 (0.270-0.795) and 0.547 (0.279-0.792), respectively]. In contrast, compared with NLR+SIRI alone, the RPA model did not provide statistical incremental value. The IDI values were 0.014 (−0.023-0.050) and 0.009 (−0.030-0.048) for OS at 3 and 5 years, and −0.025 (−0.078-0.024) and −0.037 (−0.092-0.016) for PFS.

### Subgroup analyses of RPA

**Figure 6:**
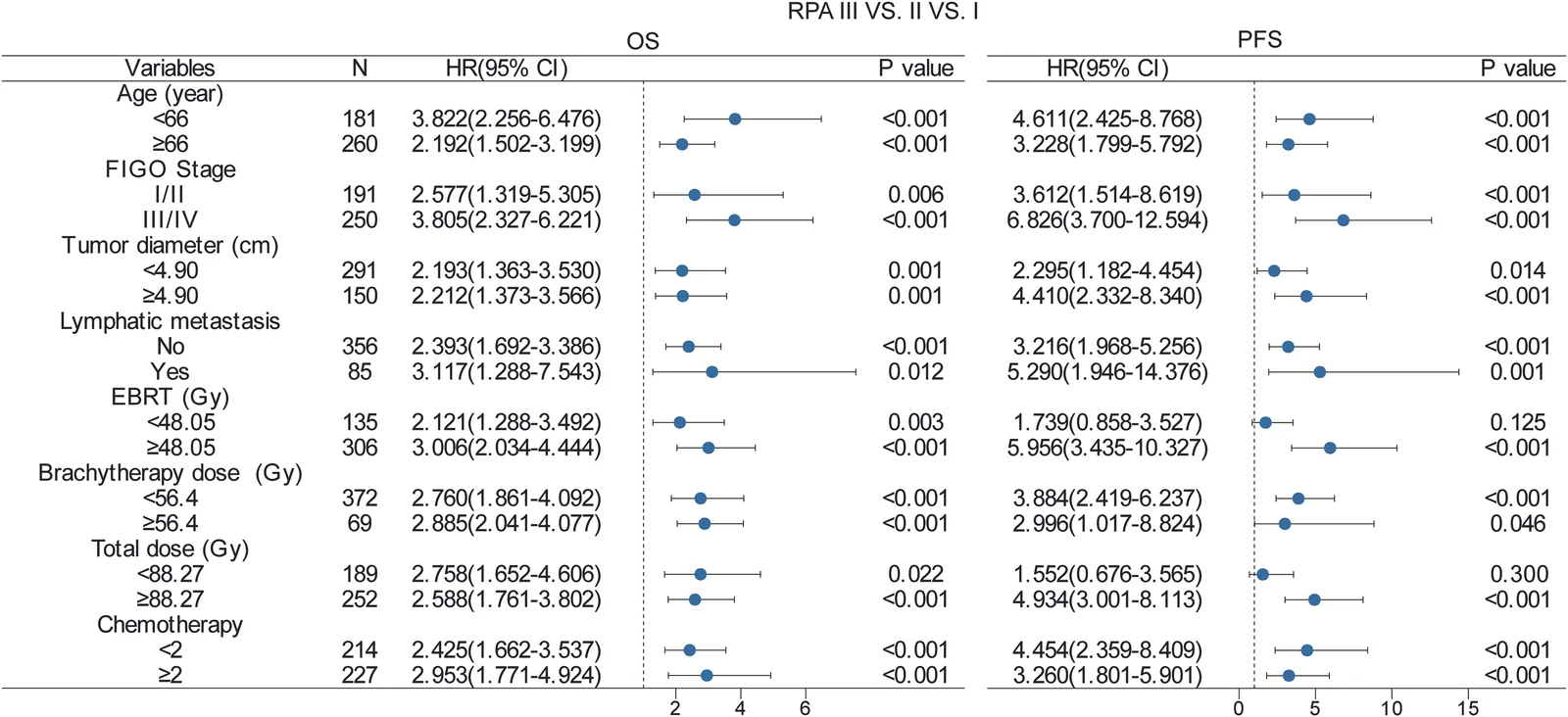
Subgroup analysis of RPA model.

Subgroup analysis demonstrated that, in terms of OS, the RPA high-risk group had a significantly higher risk of death than the low-risk group across all subgroups [all (P < 0.001)], with hazard ratios exceeding 2 in most subgroups, further highlighting the model’s strong ability to identify poor prognosis. Consistent results were observed for PFS, where patients in the high-risk group also exhibited a significantly increased risk of disease progression (**Figure 6**). Overall, the subgroup analysis suggested that the RPA risk stratification model based on NLR, SIRI, and FIGO stage appeared to maintain prognostic discriminatory ability across subgroups defined by age, FIGO stage, lymph node status, tumor size, and different treatment strategies, suggesting potential stability and clinical utility.

## Discussion

This study, based on 441 elderly patients with locally advanced CSCC who received dCRT, systematically evaluated the prognostic value of pretreatment peripheral blood inflammatory biomarkers and innovatively constructed a RPA risk stratification model integrating NLR, SIRI, and FIGO stage. The main findings are as follows: (1) NLR and SIRI were independent prognostic factors for OS and PFS, and their effects on prognosis exhibited a linear association; (2) the RPA model based on NLR, SIRI, and FIGO stage demonstrated superior predictive performance compared to the traditional FIGO staging system, enabling more precise identification of high-risk patients; and (3) the RPA model showed discriminative ability across different subgroups, including age, FIGO stage, lymph node status, and treatment strategies.

Systemic inflammatory response plays a critical role in tumor initiation, progression, and therapeutic resistance. Neutrophils can promote tumor invasion and metastasis by secreting matrix metalloproteinases that degrade the extracellular matrix, and by releasing vascular endothelial growth factor to drive tumor angiogenesis (16, 17). Monocytes are recruited to the tumor microenvironment by chemotactic factors and differentiate into tumor-associated macrophages (TAMs). Among these, M2-polarized TAMs secrete immunosuppressive factors such as IL-10, TGF-β, and prostaglandin E2, which inhibit the proliferation and cytotoxic function of CD8^+^ T cells and induce the accumulation of regulatory T cells, thereby establishing an immunosuppressive network (18, 19). Lymphocytes, particularly cytotoxic T lymphocytes and natural killer cells, are the core effectors of antitumor immunity, and their reduction directly reflects impaired host immune function (20–22). Therefore, NLR and SIRI are comprehensive indicators that can more fully and sensitively reflect the imbalance between “pro-tumor inflammation” and “antitumor immunity.” High levels of NLR and SIRI signify enhanced neutrophil-mediated pro-inflammatory and pro-invasive effects, deepened monocyte/macrophage-dominated immunosuppression, and weakened lymphocyte-mediated antitumor activity. Together, these factors synergistically accelerate tumor progression and promote treatment resistance.

The above biological rationale is strongly supported by extensive clinical evidence. Mizunuma et al. demonstrated that the pretreatment NLR serves as a significant prognostic indicator for predicting the efficacy of radiotherapy and concurrent chemoradiotherapy in patients with CC. Compared with patients presenting a high NLR, those with a low NLR exhibited a higher complete response rate and more favorable long-term survival outcomes (23). Another meta-analysis encompassing 34 studies with a total of 10,246 patients with CC systematically confirmed that high NLR predicted poor OS (24). Regarding SIRI, Chao et al. demonstrated that a higher SIRI was significantly associated with lymphovascular invasion, advanced FIGO stage, and poor prognosis, and its predictive accuracy was superior to that of PLR and LMR. Compared with the traditional FIGO staging system, the nomogram incorporating SIRI provided a more objective and reliable prediction of postoperative survival in patients with cervical cancer (25). The prognostic value of SIRI has also been validated in other solid tumors. For example, Tang et al. identified SIRI as a significant independent predictor of early disease progression in advanced NSCLC patients receiving PD-1 inhibitor therapy, and it may help identify those patients who are likely to benefit from PD-1 inhibitor treatment in routine clinical practice (26). Similarly, Ding et al. confirmed SIRI as an independent adverse prognostic factor in colorectal cancer (27). These findings are highly consistent with the results of the present study and collectively reinforce the clinical utility of NLR and SIRI as practical prognostic biomarkers across different cancer types and treatment modalities, suggesting that they may reflect a common pathway of immune-inflammatory imbalance within the tumor microenvironment.

It is noteworthy that the inflammatory biomarkers in this study were only measured at a single pretreatment time point. However, the dynamic immune-inflammatory status during chemoradiotherapy is equally critical for prognosis. Previous studies have demonstrated that longitudinal monitoring during treatment may reveal more timely prognostic information (28, 29). For instance, radiotherapy-induced tumor cell death can release antigens, triggering immune activation that manifests as a transient increase in lymphocyte count (30). Conversely, sustained inflammatory stimulation or immunosuppression may be reflected by persistent elevations in NLR or SIRI, indicating treatment resistance or poor prognosis. Therefore, extending single baseline measurements to a longitudinal time-series profile and constructing predictive models based on dynamic changes could further improve prognostic accuracy, which represents an important direction for future research.

Although NLR and SIRI possess prognostic value, single biomarkers have limited predictive capacity for complex clinical outcomes. Therefore, this study innovatively integrated NLR, SIRI, and FIGO stage to construct a RPA risk stratification model. This model classified patients into three risk groups, with 5-year OS rates of 82.1%, 70.0%, and 28.6%, respectively, demonstrating significantly superior discrimination compared to the traditional FIGO staging system (I/II stage vs. III/IV stage: 79.5% vs. 65.9%). The C-index of the RPA model was 0.625, and its AUC values for predicting 3-, 5-, and 8-year OS and PFS were all higher than those of FIGO staging or single inflammatory biomarkers. DCA also confirmed its greater clinical net benefit. The core advantage of this model lies in combining systemic inflammatory status (reflecting tumor biological behavior) with anatomical staging, enabling a comprehensive risk assessment from “anatomy” to “biology.” It exhibited stable discriminative ability across multiple key clinical subgroups, including age and lymph node status. Furthermore, as it is based on routine blood tests and staging examinations, it is simple, readily available, and facilitates broad clinical applicability. In terms of clinical value, this model helps identify extremely high-risk patients to guide more aggressive treatment (e.g., combined immunotherapy or intensified follow-up), while also identifying low-risk patients to avoid overtreatment and unnecessary toxicity.

Nevertheless, this study has several limitations. First, as a retrospective, single-center study, potential selection bias and information bias are difficult to completely avoid, which may limit the generalizability of our findings. Second, although internal validation was performed using bootstrap resampling and cross-validation, an independent external validation cohort is lacking; thus, the applicability of this RPA risk stratification model to broader populations requires further verification in multicenter, multi-regional external cohorts. Third, due to the retrospective nature of the data, comprehensive geriatric assessment parameters (e.g., Charlson Comorbidity Index, KPS/ECOG performance status, and Geriatric Nutritional Risk Index) were not routinely documented and therefore could not be incorporated into the competing models; moreover, the RPA model exhibited only moderate discrimination (C-index of 0.625 for OS) and its incremental prognostic value over NLR–SIRI alone was limited (as reflected by non-significant IDI and NRI). Consequently, the model should be viewed as an auxiliary stratification tool rather than a standalone instrument for guiding treatment decisions. Fourth, only pretreatment peripheral blood inflammatory biomarkers at a single time point were analyzed; dynamic changes during treatment were not captured, making it difficult to fully assess the impact of temporal fluctuations in systemic inflammatory status on patient prognosis.

## Conclusion

Pretreatment NLR and SIRI are independent prognostic biomarkers for OS and PFS in elderly CSCC patients receiving dCRT. Integrating them with FIGO stage via an RPA model enables superior risk stratification. This model identifies three distinct prognostic groups, guiding personalized treatment decisions. Future multicenter studies with longitudinal monitoring are needed to validate these findings.

## Data Availability

The raw data supporting the conclusions of this article will be made available by the authors, without undue reservation.
